# A Method for 3D Building Individualization Integrating SAMPolyBuild and Multiple Spatial-Geometric Features

**DOI:** 10.3390/s26030999

**Published:** 2026-02-03

**Authors:** Lianshuai Cao, Yi Cheng, Zheng Zhang, Ge Zhu, Kunyang Ma, Xinyue Xu

**Affiliations:** Institute of Geographic Spatial Information, PLA Information Engineering University, Zhengzhou 450001, China; cliansy230@163.com (L.C.); giser_zzy@infu.ac.cn (Z.Z.); 15138698555@163.com (G.Z.); gisermky@126.com (K.M.); 13382188144@163.com (X.X.)

**Keywords:** 3D building individualization, SAMPolyBuild, spatial-geometric features, Jensen-Shannon divergence, Earth Mover’s Distance

## Abstract

**Highlights:**

**What are the main findings?**

- A novel framework integrating SAMPolyBuild’s zero-shot capability with spatial-geometric feature refinement achieves high-precision 3D building individualization without pre-training.
- The joint JS-EMD metric effectively identifies building-ground interfaces by quantifying spatial distribution shifts in normal vector angles.

**What are the implications of the main findings?**

- The method provides a lightweight, efficient solution for large-scale urban modeling, reducing reliance on manual effort and extensive training data.
- It establishes a zero-shot learning paradigm that effectively transfers 2D foundation model segmentation capabilities to 3D spatial analysis.

**Abstract:**

Individualization of buildings is one of the key issues in the establishment of three-dimensional (3D) building models. Most existing individualization methods rely on inefficient manual separation, while deep learning approaches require extensive pre-training and are highly influenced by the spatial structure of the models. To address these issues, this paper proposes a novel method for 3D building individualization that integrates SAMPolyBuild with multiple spatial-geometric features. Leveraging the zero-shot learning capability of SAMPolyBuild, the method first performs coarse extraction of individual buildings, then refines the extraction accuracy using multiple spatial-geometric features. Innovatively, two statistical parameters—Jensen-Shannon Divergence and Earth Mover’s Distance—are introduced into the building identification process. To validate the feasibility and effectiveness of the proposed method, experiments were conducted on the Semantic Urban Meshes (SUM) dataset. The results demonstrate that the method can effectively extract individual building models from urban oblique photogrammetric 3D models, achieving an F1-score of approximately 0.83 for buildings with typical spatial structures.

## 1. Introduction

In the construction of real-world 3D city models, oblique photogrammetric 3D building models serve as core urban components and often represent a key focus of development efforts [1,2]. In contemporary practice, such models are primarily generated using photogrammetric techniques [3,4] or LiDAR point cloud technology [5,6] to synchronously capture data from multiple viewpoints [7]. Nevertheless, models produced through these methods form composite data structures consisting of continuous triangular mesh networks correlated with texture data [8]. This inherent characteristic complicates the individualization and semantic management of real-scene 3D representations. To achieve entity-based management of urban elements, it is essential to extract individualized components from 3D models [9,10].

Presently, acquiring individual building models mainly relies on cadastral data to obtain vector boundaries [11]. In practical production workflows, manual operation—often involving specialized software such as DP-Modeler 2.3 for manual segmentation—remains necessary. Although manual extraction ensures high precision in model isolation, it consumes substantial human and time resources, resulting in notably low efficiency for large-scale engineering projects [12]. In recent years, with the rapid advancement of cutting-edge technologies such as deep learning and artificial intelligence, automatic individualization techniques based on semantic segmentation model training have gradually emerged [13]. Compared to traditional manual approaches, these techniques offer significantly improved efficiency. The primary methods for semantic extraction involve converting the model into point cloud or image formats [14]. For example, Qi et al. introduced the PointNet++ model, which notably enhanced the fine-grained recognition capability and robustness of point cloud processing [15]. Rong et al. proposed a large-scale urban scene reconstruction method based on active learning [16]. Additionally, there are approaches specifically designed for triangular mesh learning, such as the PSSNet deep learning framework developed by Gao, which is applied to semantic segmentation of large-scale urban texture meshes [17]. Although deep learning-based recognition methods demonstrate high efficiency, they require substantial computational resources during the initial model training stage. Furthermore, challenges remain in accurately recognizing complex scenes such as dense urban blocks and irregularly shaped buildings, often necessitating manual review and correction in post-processing stages.

To address the aforementioned issues—particularly the inefficiency of manual methods, the data dependence of deep learning approaches—this paper proposes a novel 3D building individualization method that integrates SAMPolyBuild [18] zero-shot segmentation with a ground data recognition method based on two-stage spatial-geometric features, whose core lies in the joint use of the Jensen-Shannon (JS) divergence and Earth Mover’s Distance (EMD) metrics. By analyzing the normal vector distributions of horizontal slices extracted from 3D model data, this method achieves precise detection of the building-ground interface. This design comprehensively leverages the zero-shot training capability of the SAM-derived SAMPolyBuild model [19] (reducing reliance on labeled training data) and the robustness of spatial-geometric features [20] in describing structural differences between buildings and the ground. The synergy between JS divergence and EMD effectively captures the spatial directional variations in model structures. To a certain extent, this method reduces the dependence on pre-trained deep learning models during the 3D building individualization process while maintaining a satisfactory level of extraction accuracy for both regular and irregular building structures. Experimental results demonstrate that the proposed method achieves high recognition accuracy without the need for sample-based training, providing an efficient and reliable solution for large-scale 3D building individualization.

## 2. Materials and Methods

**Figure 1:**
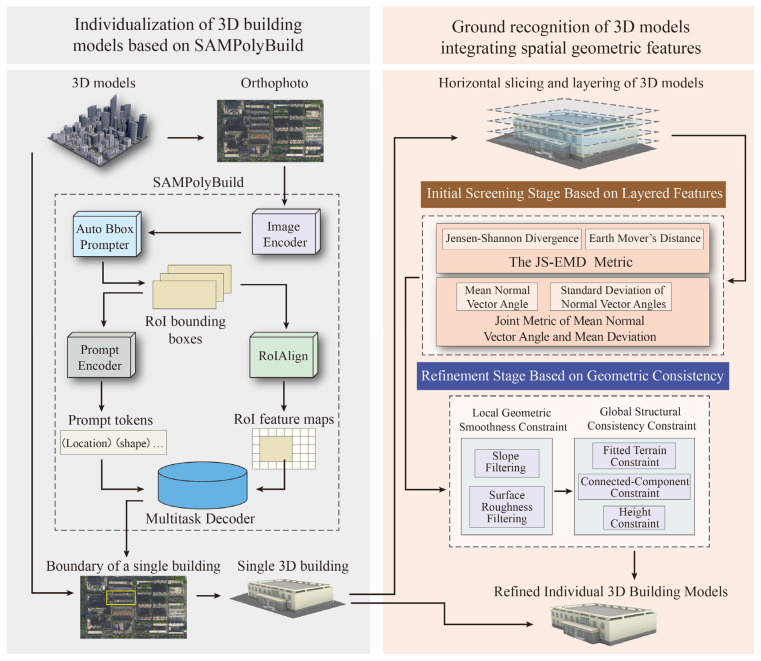
Schematic diagram of the overall methodology.

The individualization method proposed in this paper consists of two main stages (Figure 1). In the first stage, an orthophoto of the 3D model area is initially acquired. The SAMPolyBuild model is then employed to identify building boundaries within this orthophoto, yielding precise building boundary information. Subsequently, this extracted boundary information is used to segment the original 3D building model, resulting in preliminary individual building models. Nonetheless, as the boundary data is derived from an orthographic view, the building base information may be partially obscured, leading to the inclusion of redundant ground data in the initial models.

Consequently, in the second stage, building upon the initially individualized models, redundant ground data is removed. This is achieved by leveraging the vertical variations in multiple spatial-geometric features of the building models to further identify and eliminate ground data. After the removal of this ground data, refined and highly accurate individual building models are obtained, thus achieving precise segmentation.

### 2.1. Individualization of 3D Building Models Based on SAMPolyBuild

The SAMPolyBuild model, developed by Wang et al. [18] based on the Segment Anything Model (SAM), is a segmentation model specifically designed for polygonal building extraction. Its methodology integrates Gaussian vertex map prediction with a mask-guided vertex connection algorithm.

**Figure 2:**
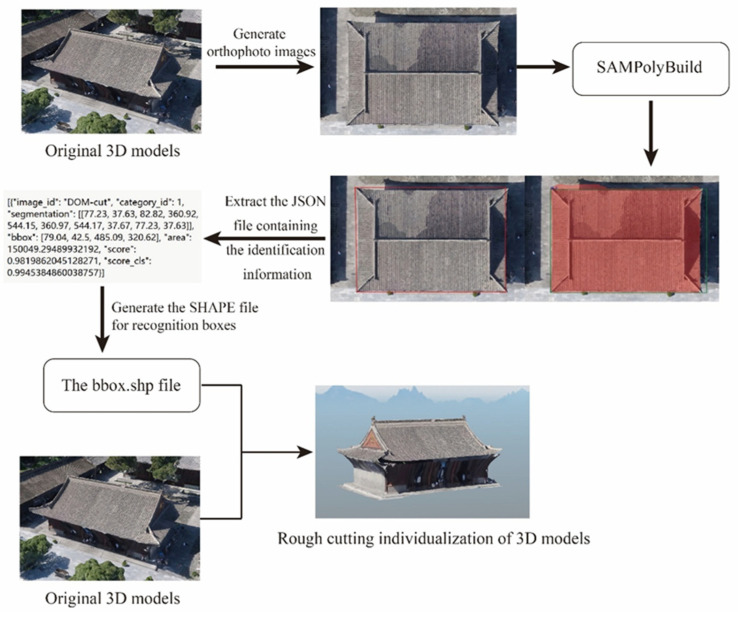
Workflow for 3D building individualization based on the SAMPolyBuild.

In this study, the oblique photogrammetric 3D model of the Dayan Pagoda scenic area in Xi’an was selected as the test site. The SAMPolyBuild model was applied to extract buildings across the entire scenic area. First, the original image was processed through the SAM image encoder to output a feature map. A Faster R-CNN-based automatic bounding box prompter then generated building bounding boxes with center prompt points. Subsequently, the generated boundaries underwent a refinement process. A prompt encoder generated prompt tokens for the bounding boxes, which were then encoded into prompt embeddings. Meanwhile, RoIAlign was utilized to extract Region of Interest (RoI) features for individual buildings from the feature map. Both the prompt embeddings and the RoI features were fed into an extended SAM multi-task decoder to simultaneously predict the mask, Gaussian vertex map, and boundary map. Finally, candidate vertices were filtered using a mask-guided vertex connection algorithm, and the polygon was simplified to output the vectorized building boundaries. Based on the acquired boundary data, spatial-geometric cutting was performed on the 3D model to generate individualized 3D building models (Figure 2).

**Figure 3:**
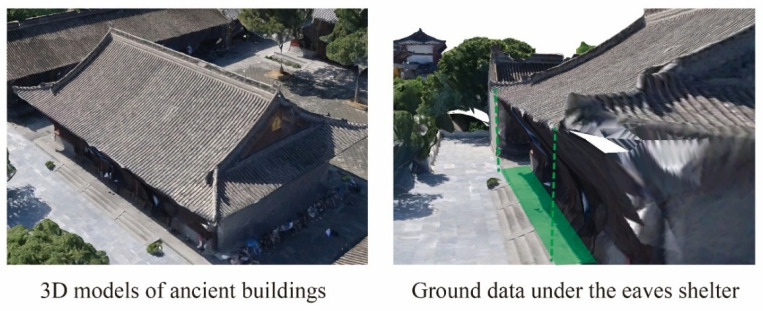
Ground artifacts caused by eave occlusion.

By extracting building boundaries from orthophotos, a certain degree of effective horizontal segmentation of buildings can be achieved. However, in practical applications, many buildings exhibit a morphological characteristic described as “wider at the top and narrower at the bottom,” where wide eaves extend outward from relatively narrow building bodies to provide protection against natural precipitation such as rain and snow. Relying solely on orthophotos for segmentation in such cases often results in the retention of ground data beneath the eaves, thereby compromising the accuracy of building individualization. For instance, in the study area of this research, buildings dating back to the Ming and Qing dynasties feature typical official-style architectural designs, as illustrated in Figure 3. These structures commonly include single-eaved hard hill roof or overhanging hill roof style, with eaves that extend outward from the façades to a significant degree. As a result, the aforementioned method alone proves insufficient for accurately removing ground data located beneath the eaves.

To achieve precise cutting of architectural models, it is crucial to identify and remove the ground data within the target region. In light of this, we analyzed the spatial-geometric characteristics of both buildings and the ground in 3D models. Based on this analysis, we developed a ground recognition method for 3D models that integrates multiple spatial-geometric features. This method facilitates more accurate segmentation and cutting of architectural components within 3D models.

### 2.2. Ground Recognition of 3D Models Integrating Spatial-Geometric Features

In urban oblique photogrammetric 3D models, buildings and the ground exhibit distinct spatial-geometric distribution characteristics [21]. Numerous scholars have focused on achieving target recognition by quantifying various spatial parameters of 3D models. For example, Xu et al. extracted individual buildings from 3D mesh data using parameters such as position, area, and geometric deviation [22]. Shah et al. accomplished target recognition of 3D structures by leveraging spatial structural features, including the distance between a key point and its two nearest neighbors, as well as transformation matrices [23]. Typically, buildings are characterized by a wide range of elevation values, with the triangular meshes representing building façades predominantly exhibiting vertical distribution patterns. In contrast, the ground surface generally displays a smoother and more horizontally extended form, typically located at lower elevations within the model. Theoretically, semantic recognition of buildings and the ground can be achieved based on elevation and vertical relationships. However, in practice, errors during data acquisition, limitations in algorithmic precision, and the complexity and variability of real-world environments often result in non-vertical or non-approximately vertical connections between buildings and the ground in the generated models [24]. Additionally, the presence of objects such as vegetation and vehicles can further complicate the accurate identification of spatial orientation relationships. To address these challenges, this study proposes a two-stage ground data extraction method integrating multiple spatial-geometric features, which comprises a hierarchical preliminary screening stage based on the JS-EMD (Jensen–Shannon Divergence and Earth Mover’s Distance) index, mean normal vector angle, and neighborhood angle standard deviation, followed by a geometric consistency refinement stage utilizing features such as slope, roughness, and connectivity to achieve accurate extraction of ground data.

#### 2.2.1. Preliminary Screening Stage Based on Hierarchical Features

##### The Joint JS-EMD Metric

In 3D models generated through oblique photography, the boundary lines between buildings and the ground typically exhibit horizontally extended spatial characteristics. By analyzing the spatial positioning of these boundary lines, it becomes feasible to identify and eliminate redundant ground face data associated with buildings. Given their horizontally extending features, this paper proposes a method of uniformly slicing the model at different horizontal levels to examine the distinct spatial-geometric properties of each layer. By analyzing variations in relevant feature indicators across layers, the spatial position of the interface can be effectively explored. As previously mentioned, the presence of noise errors—such as those caused by vegetation and vehicles—near the interface may compromise accuracy if the interface is determined solely based on the vertical relationships of triangular facets. However, from an overall model perspective, most triangular facets near the interface still demonstrate a spatial pattern of gradual transition from horizontal to vertical orientation [25]. To capture these structural variations, this paper conducts a statistical analysis of the normal vector angle distributions of triangular facets across elevation layers. A histogram is employed to represent the distribution of triangular facets within each elevation layer, with the horizontal axis indicating the normal vector angle and the vertical axis representing the frequency of faces within each angular range. To further validate the effectiveness of the proposed method, a case study is conducted using a single building located in the central area of Helsinki, Denmark. This model retains not only the building and ground data but also includes elements such as vegetation and vehicles at the interface, aligning with the preset conditions of the experimental setup.

**Figure 4:**
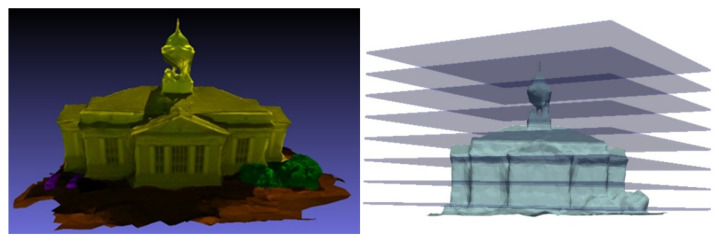
Schematic diagram of a 3D oblique photogrammetric building model and its horizontal slicing.

**Figure 5:**
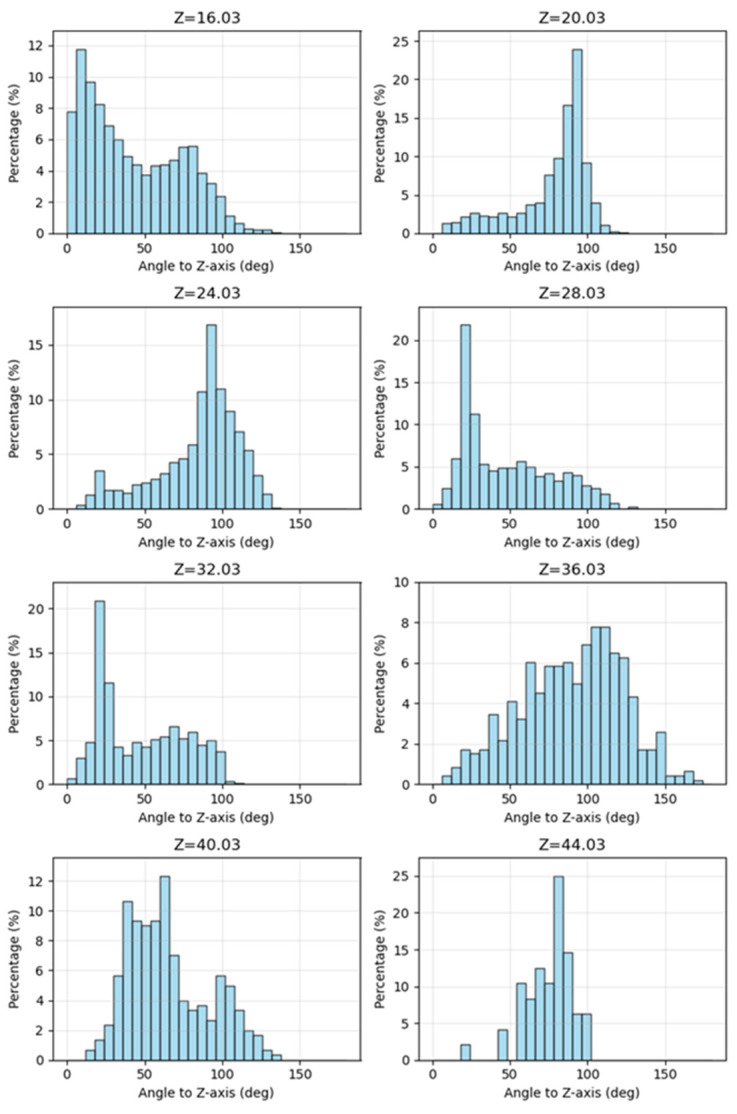
Histogram of normal vector angle distributions across different cutting layers.

Based on the aforementioned analysis, the model underwent a horizontal slicing operation utilizing a vertically equidistant and horizontally stratified methodology. Subsequently, a statistical analysis was conducted on the distribution of normal vectors from the triangular facets within each layer. As illustrated in Figure 4, the elevation range of the model building spans approximately from 14 to 44 units. For the purpose of statistical evaluation, the model was uniformly divided into strata at an elevation interval of 4 units. The findings are summarized in Figure 5, where the title elevation value of each histogram denotes the central elevation of the respective layer. The horizontal axis represents the normal vector angle, segmented at intervals of 6°, while the vertical axis indicates the proportion of triangular facets within each angular range. As observed from the stratification depicted in Figure 4, the slicing layers, from bottom to top, correspond to histograms with progressively increasing central elevation values. These histograms are labeled sequentially from 1 to 8. Histogram No. 1 illustrates the statistical results of the slicing layer encompassing the ground surface. Upon examination, the majority of the data in this layer consists of horizontal or near-horizontal triangular facets on the ground, which is reflected in the histogram as a high proportion of low-angle normal vectors. A smaller portion corresponds to triangular facets located on vertical or near-vertical building façades, which is represented in the histogram by a relatively low proportion of normal vectors near 90°. Consequently, the distribution of normal vector angles serves as an effective indicator of the spatial characteristics of triangular facets within each slicing layer.

Similarly, the second histogram illustrates the distribution of normal vector angles corresponding to the triangular facets within the sub-bottom layer of the model, as depicted in the cross-sectional view of Figure 4. The sub-bottom layer is predominantly composed of building façades; consequently, the histogram exhibits a significant proportion of facets with normal vector angles around 90°, particularly within the range of 84° to 96°, which accounts for nearly 45% of the total. A majority of the vegetation data in the model is concentrated in this layer. Vegetation structures are generally ellipsoidal in shape, and their surface facets exhibit a smooth transition from a vertical to a horizontal orientation, resulting in a relatively uniform distribution of normal vector angles. While the distribution of acute angles appears relatively even across the histogram, this does not significantly influence the overall distribution characteristics of the layer. By analyzing the peak positions, peak heights, and degree of concentration in the first and second histograms, it can be observed that the triangular facets demonstrate a trend of transitioning from a predominantly horizontal orientation to a more vertically concentrated arrangement. This change reflects the model’s transition from a relatively flat ground surface to a building’s façade. Histograms 3 through 8 can also reflect the spatial structural characteristics of the corresponding model sections. Therefore, by analyzing the variation patterns in adjacent histograms, the spatial features of the entire model along the Z-axis can be effectively captured. Histograms 1 and 2 exhibit significant changes in peak positions and concentration levels, making the structural variations relatively easy to interpret. In contrast, histograms 2 and 3 display similar peak positions and concentration levels, which makes it challenging to distinguish structural differences between them. To provide a more precise description of the degree of change between adjacent histograms, we introduce two quantitative metrics—Jensen-Shannon Divergence and Earth Mover’s Distance—for the analysis of such variations.

(1) The Jensen–Shannon Divergence

The Jensen-Shannon (JS) divergence, also known as the Jensen-Shannon distance, is a symmetric statistical measure used to assess the similarity between two probability distributions [26]. It is based on the Kullback–Leibler (KL) divergence, which quantifies the relative difference between two probability distributions defined over the same event space X. The KL divergence between two probability distributions p(x) and q(x) is mathematically defined as:

(1) $$KL(p||q)=\sum_{x\in X}p(x)\log\frac{p(x)}{q(x)}$$

The KL divergence is not symmetric in nature [27]. In contrast, the JS divergence overcomes this limitation by providing a symmetric measure, making it more appropriate for quantifying the dissimilarity between two probability distributions. The JS divergence is formally defined as:

(2) $$JSD(P||Q)=\sum_{x\in X}\left(p(x)\log\frac{2p(x)}{p(x)+q(x)}+q(x)\log\frac{2q(x)}{p(x)+q(x)}\right)$$

**Figure 6:**
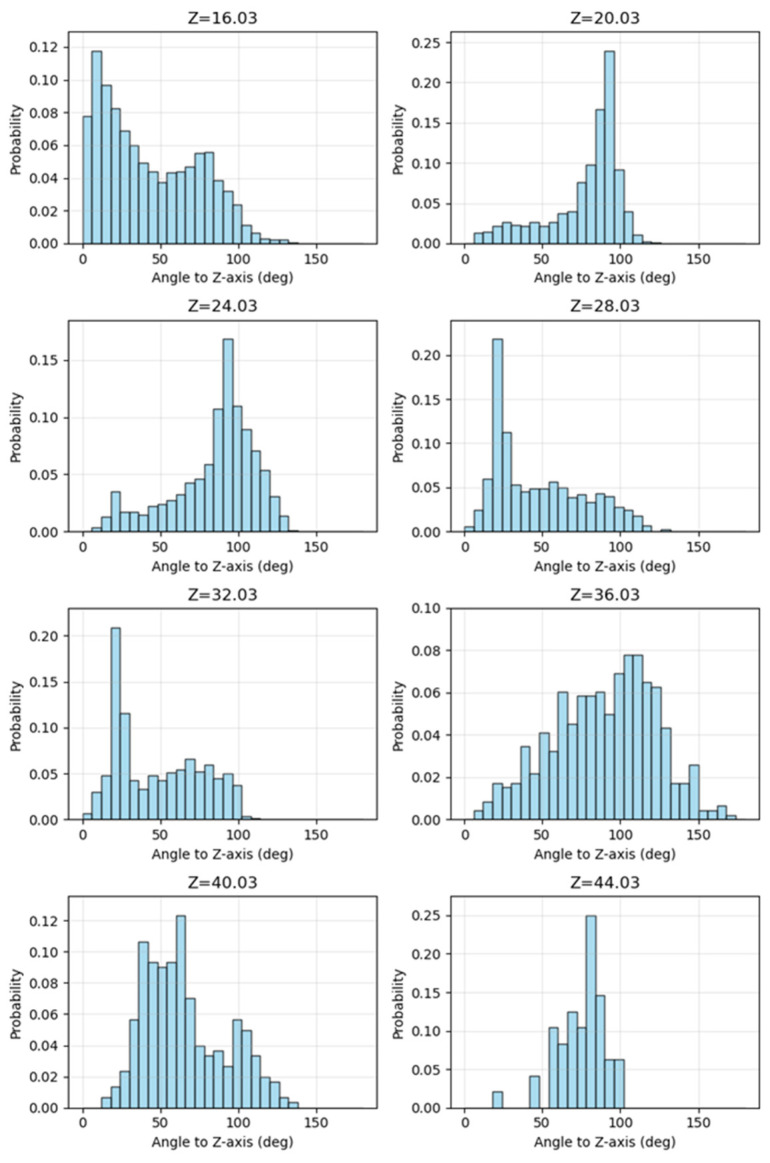
Histogram of probability distributions for normal vector angles across different cutting layers.

Although JS divergence originates from information theory, it has been extensively applied across various disciplinary fields. In natural language processing, it is employed to compare word frequency distributions across different texts [28]; in image analysis, it aids tasks such as image classification by measuring the similarity between color histograms [29]. In this study, the JS divergence is applied to quantify the difference in the distribution of normal vector angles across adjacent cutting layers of the model. Since the JS divergence is fundamentally designed to measure differences between probability distributions, it is essential that the histogram of normal vector angle distributions in each cutting layer conforms to a probability distribution, where the total area sums to 1 [30]. Consequently, a normalization process must be applied to transform the histogram into a valid probability distribution format compatible with the JS divergence metric. The results are shown in Figure 6 and Table 1.

Based on the analysis of histogram shapes and the calculated JS divergence values, the distribution patterns of histogram 1 and histogram 2 demonstrate significant differences, as evidenced by a relatively high JS divergence value of approximately 0.18. In contrast, the distributional difference between histogram 2 and histogram 3 is minimal, with a corresponding JS divergence value of approximately 0.06. This pattern remains consistent in the analysis of subsequent histograms. By utilizing JS divergence as a quantitative measure, we have effectively evaluated the differences in the distribution of normal vector angles across adjacent cutting layers. Specifically, a higher JS divergence value indicates a more pronounced change in the spatial structure of the model between adjacent layers.

(2) Earth Mover’s Distance

In previous studies, JS divergence, as an information-theoretic metric, has been effective in capturing the overall differences in the distribution of normal vectors between adjacent cutting layers. However, it fundamentally operates as a point-wise comparison based on probability distributions, primarily measuring the overlap and divergence within individual bins of the distributions [31]. Its sensitivity to shifts in the positions of distribution peaks is relatively low. Consequently, when the histograms of normal vectors from two adjacent layers exhibit similar shapes but experience a shift in peak positions, JS divergence may still yield a small value, potentially overlooking significant changes in the overall distribution pattern. To address this limitation, this paper introduces the Earth Mover’s Distance (EMD), a metric specifically designed to quantify morphological shifts in probability distributions [32]. The conceptual basis of EMD lies in treating each probability distribution as a mass distribution and computing the minimum total cost required to transform one distribution into another. This cost is determined by the product of the transported mass and the distance over which it is transported. In contrast to the JS divergence, which relies on point-to-point distance metrics, EMD accounts for the spatial displacement of probability mass. As such, it is particularly effective in capturing spatial movement and shape variations in the peaks of normal vector distributions across adjacent segmentation layers. This makes EMD especially suitable for characterizing continuous changes in the normal vector angle histogram [33]. For the specific case of normal vector angle distribution histograms considered in this paper, EMD is defined as follows [34]: Let the histograms of the normal vector angle distributions for the k-th and (k+1)-th cutting layers be represented as probability distribution vectors, respectively:

(3) $$P^{\left(k\right)}=\{p_{i}{\}}_{i=1}^{n},Q^{\left(k+1\right)}=\{q_{j}{\}}_{j=1}^{n},\sum_{i=1}^{n}p_{i}=\sum_{j=1}^{n}q_{j}=1$$

where n is the number of angular bins. $p_{i}$ and $q_{j}$ are the probabilities that the normal vector’s angle falls into the i-th and j-th angular bin, respectively. A distance matrix $D=[d_{ij}{]}_{n\times n}$ is defined, where each element $d_{ij}$ is the ground distance between the corresponding bins, taken as the absolute angular difference: $d_{ij}=|c_{i}-c_{j}|$, with $c_{i}$ and $c_{j}$ being the central angles of the i-th and j-th bins, respectively. The EMD is computed by solving the following linear programming problem for the optimal flow matrix $F=[f_{ij}]:$

$$EMD(P^{\left(k\right)},Q^{\left(k+1\right)})=\underset{F}{min}\sum_{i=1}^{n}\sum_{j=1}^{n}f_{ij}d_{ij}$$

$$\mathrm{s.t.}\;\;\sum_{j=1}^{n}f_{ij}=p_{i},\;i=1,2,\cdots ,n$$

$$\sum_{i=1}^{n}f_{ij}=q_{j},\;j=1,2,\cdots ,n$$

$$\sum_{i=1}^{n}f_{ij}=q_{j},\;j=1,2,\cdots ,n$$

(4) $$f_{ij}\geq 0,\;\forall i,j$$

Following the aforementioned formula, the EMD was calculated for the normal vector angle histograms of each adjacent cutting layer pair. The computed EMD values are summarized in Table 2.

By comparing the histogram with the EMD value, it can be observed that the peak values of histogram 1 and histogram 2 exhibit significant differences, resulting in a relatively large EMD value of 34.67. In contrast, histogram 2 and histogram 3 display similar peak values, corresponding to a much smaller EMD value of only 7.39. Through a comparative analysis of the model’s cutting layers, it is evident that the transition from cutting layer 1 to cutting layer 2 involves a structural change from ground to façade, accompanied by a shift in the dominant direction of the normal vectors of the triangular facets from horizontal to vertical. On the other hand, when transitioning from cutting layer 2 to cutting layer 3, both layers predominantly contain façade data from the same building, resulting in minimal change in the primary direction of the normal vectors. The EMD values between subsequent cutting layers demonstrate a consistent relationship with the spatial structural characteristics of the model. Therefore, by computing the EMD values between adjacent cutting layers, it is possible to effectively capture the variations in the dominant directions of the normal vectors across these layers.

(3) The Joint Evaluation Method for JS-EMD

In the aforementioned analysis of building instances, JS divergence and EMD values were effectively employed to accurately characterize the evolving trends in the histogram of normal vector angle distributions across adjacent cutting layers. These quantitative metrics enable further inference regarding the evolution of the spatial structure of the building model. As evidenced by the analysis, both JS divergence and EMD values increase significantly when substantial changes occur in the spatial structure; however, they capture different characteristics. The proposed method leverages JS divergence, which is based on changes in the entropy information of probability distributions, to focus on absolute differences within the same angular interval. This reflects abrupt changes in the proportion of triangular facets within that interval, making it particularly suitable for detecting localized, fault-like structural variations. In contrast, the EMD value represents the morphological displacement distance of the probability distribution in space, thereby capturing overall distribution changes and showing heightened sensitivity to shifts in peak positions. In oblique photogrammetric 3D models, as triangular facets transition from ground to building surfaces, their orientation shifts from horizontal to vertical. This transition manifests in the angle distribution histogram as a peak shift from approximately 0° to approximately 90°, resulting in a relatively large EMD value. In the actual model, the junction between the ground and buildings is typically represented by small-area inclined surface facets. Specifically, during the transition from ground to façade, the normal angle distribution across successive layers often exhibits a smooth process characterized by “continuous peak drift with approximately unchanged shape.” For evaluating differences between such adjacent layers, the JS divergence shows a second-order small response to minor displacements, often remaining below the threshold in the early stages of the transition. Meanwhile, when the distributions barely overlap, JS divergence tends to saturate, making it difficult to reflect the magnitude of displacement. In contrast, the distance-based EMD value responds nearly linearly to displacement, enabling earlier and more stable detection of the continuous drift from 0° to 90°. Therefore, this paper adopts a joint criterion combining JS divergence and EMD value: EMD value provides the magnitude of displacement, while JS divergence confirms shape deviation. Only when both parameters exceed their respective thresholds is a significant change between segmentation layers identified. This design both captures the starting point of the transition earlier and avoids false alarms caused by minor distant peaks detected solely by EMD value.

##### The Joint Metric of the Mean and Standard Deviation of Normal Vector Angles

In Section The Joint JS-EMD Metric, we employed the JS divergence and EMD value as combined metrics to detect the boundary between the ground and buildings. However, as relative metrics, they are limited to measuring variations between consecutive slicing layers and can only indicate that the interface is in proximity to the slicing layer that satisfies the threshold criteria, without identifying the absolute spatial structure of the triangular facets within each layer. For instance, using the joint JS divergence and EMD metrics, we are able to capture structural transitions from building façades to roofs, yet no ground data is identified in this region. To address this limitation, we introduced the mean and standard deviation of the normal vector angles to further distinguish ground data within the identified layer.

(1) Mean Value of Normal Vector Angles

**Figure 7:**
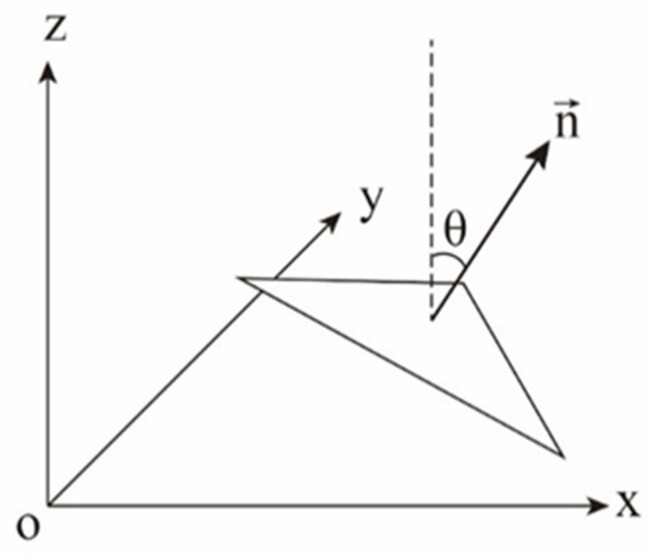
Schematic diagram of normal vector angles for a triangular facet.

The joint JS-EMD metric enables the identification of structures within the model where abrupt spatial morphological changes occur. Such structures include transitions from the ground to building façades and from building façades to rooftops, which are specifically manifested as transitions of triangular facets from a horizontal to a vertical orientation, and vice versa. In this study, the mean normal vector angle is employed to determine the ground layer data (Figure 7), which is computed as the mean of the normal vector angles of all triangular facets within that layer [35].

Suppose the candidate cutting layers identified by the joint JS-EMD metric include the L-th and (L+1)-th layers. The L-th layer contains $N_{L}$ facets, and the unit normal vectors of all facets in this layer are denoted as $n_{i}$. Given a unit vertical vector v, the angle $\theta_{i}$ between the i-th facet and the vertical direction is given by:

$$n_{i}=(n_{ix},n_{iy},n_{iz}),\;i=1,2,\ldots ,N_{L}$$

v=(0,0,1)

(5) $$\theta_{i}=\arccos(n_{i}\cdot v)=\arccos(n_{iz})$$

Therefore, the mean normal vector angle $\theta_{i}$ for the facets in the i-th layer is given by:

(6) $${\bar{\theta }}_{L}=\frac{1}{N_{L}}\sum_{i=1}^{N_{L}}\theta_{i}$$

Under ideal conditions, the normal vector angle of ground triangular facets should theoretically be 0°. However, in practical measurements, due to factors such as measurement errors and interference from other objects, this angle is not strictly 0° but exhibits irregular fluctuations [36]. Nonetheless, the majority of ground triangular facets still fall within the range of relatively small angle values. Similarly, the normal vector angles of building façade facets are primarily distributed within intervals close to 90°. Therefore, instead of relying solely on the absolute value of ${\bar{\theta }}_{L}$ for ground data identification, we compare it with the mean angle value ${\bar{\theta }}_{L+1}$ of another layer within the candidate cutting set. If the condition ${\bar{\theta }}_{L}<{\bar{\theta }}_{L+1}$ is satisfied, and the mean elevation value of the facets in these two candidate cutting layers satisfies ${\bar{h}}_{L}<{\bar{h}}_{L+1}$, the L-th layer and all data below its elevation are determined to be ground data.

(2) Standard Deviation of Normal Vector Angles

Within the candidate cutting layers, the identification of ground data in the model is determined based on the relationship between the mean normal vector angle and the mean elevation value. The primary basis for this identification lies in the fact that the layer with a smaller mean angle $\bar{\theta }$ can be characterized as representing ground facets. However, as a one-dimensional metric, $\bar{\theta }$ only reflects the overall distribution of angles and fails to effectively capture local variations in the angular distribution. This implies that under the same mean angle $\bar{\theta }$, the dispersion of the angular distribution is not uniquely determined, which may affect the validity and robustness of the aforementioned discrimination method.

Therefore, we further introduce the standard deviation of normal vector angles to constrain the dispersion of the angular distribution of the triangular facets. Compared to variance—a traditional statistic for measuring dispersion—the standard deviation shares the same unit as the data, has a smaller numerical magnitude, and more intuitively reflects actual fluctuations [37]. Thus, this study employs the standard deviation to quantify the variability in the angular distribution of triangular facets within each layer.

To more accurately represent local distribution characteristics and reduce the influence of individual outliers, we adopt a method that first computes the local neighborhood standard deviation before averaging these values to obtain the overall standard deviation for each cutting layer. Assume that cutting layer L contains a set of facets $F_{L}$. Let $n_{i}$ denote the unit normal vector of the i-th facet in the layer, and N(i) represent the set of its neighboring facets. The angle between this facet and a neighboring facet j is denoted as $\theta_{ij}$. Then, the following procedure is applied:

(7) $$\theta_{ij}=\arccos(n_{i}\cdot n_{j})$$

Mean neighborhood angle of the facet:

(8) $$\bar{\theta_{i}}=\frac{1}{\left|N(i)\right|}\sum_{j\in N(i)}\theta_{ij}$$

Local standard deviation of angles for the facet:

(9) $$\sigma_{i}=\sqrt{\frac{1}{\left|N(i)\right|}{\sum_{j\in N(i)}\left(\theta_{ij}-\bar{\theta_{i}}\right)}^{2}}$$

Global dispersion of the cutting layer (mean of local standard deviations):

(10) $$\bar{\sigma_{L}}=\frac{1}{\left|F_{L}\right|}\sum_{i\in F_{L}}\sigma_{i}=\frac{1}{\left|F_{L}\right|}\sum_{i\in F_{L}}\sqrt{\frac{1}{\left|N(i)\right|}{\sum_{j\in N(i)}\left(\theta_{ij}-\bar{\theta_{i}}\right)}^{2}}$$

Through the evaluation of the $\bar{\sigma_{L}}$ value, the candidate cutting layers that satisfy the mean normal vector angle condition are further subjected to standard deviation assessment. If the standard deviation falls within a predefined threshold range, the distribution of normal vector angles in the cutting layer can be considered smooth and concentrated. This confirms the validity of the mean angle criterion and effectively mitigates misjudgments caused by significant fluctuations in the angular distribution of the facet normal vectors, thereby enhancing the recognition accuracy of the proposed method.

##### Implementation of the Preliminary Screening Stage

In the preceding discussion, we employed a series of spatial-geometric feature parameters—including the joint JS-EMD metric, the mean normal vector angle, and the standard deviation of normal vector angles—to identify and extract ground data from the 3D model. This approach enabled the acquisition of elevation values near the building-ground interfaces within the 3D model, based on which the segmentation of buildings from the ground can be achieved.

However, in practical surveying and production contexts, the building and ground surfaces in oblique photogrammetric 3D models are not always coplanar. Due to slopes or measurement errors, the interface often exhibits an irregular spatial curve. Therefore, prior to applying the aforementioned integrated decision method, we first preprocess the data. Guided by the principle that a local segment of a curve can be approximated as a straight line, the 3D model is partitioned horizontally into regular grid cells. Within each cell, the joint JS-EMD metric is applied to detect adjacent cutting layers where abrupt spatial changes occur, designating them as candidate identification layers.

Subsequently, the mean normal vector angle and its relationship with elevation are utilized within these adjacent candidate layers to identify the ground layer. The standard deviation of normal vector angles is further employed to eliminate misjudgments caused by large fluctuations in the distribution of facet normals. This process yields the elevation value of the building-ground interface within each grid cell.

Finally, the elevation values assigned to the centers of the ground-labeled mesh faces within each grid cell are aggregated to form a set of three-dimensional sample points $\left(x_{i},y_{i},z_{i}\right)$. These points represent the spatial distribution of the detected ground surface. The surface is modeled using a quadratic polynomial function of the form:

(11) $$z=ax^{2}+by^{2}+cxy+dx+ey+f$$

where (a,b,c,d,e,f) are the polynomial coefficients describing curvature, tilt, and offset of the fitted ground surface.

**Figure 8:**
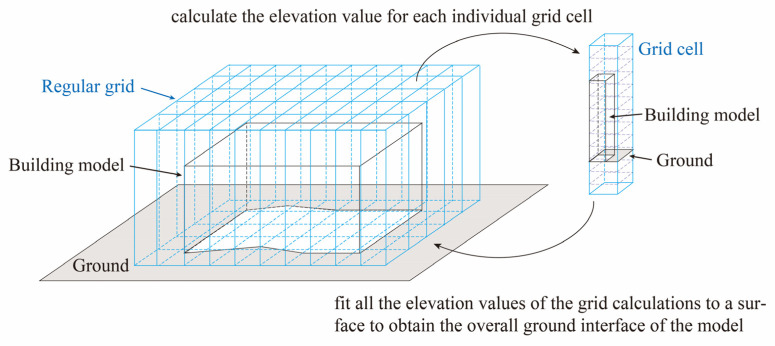
Schematic diagram of the regular grid partitioning.

**Figure 9:**
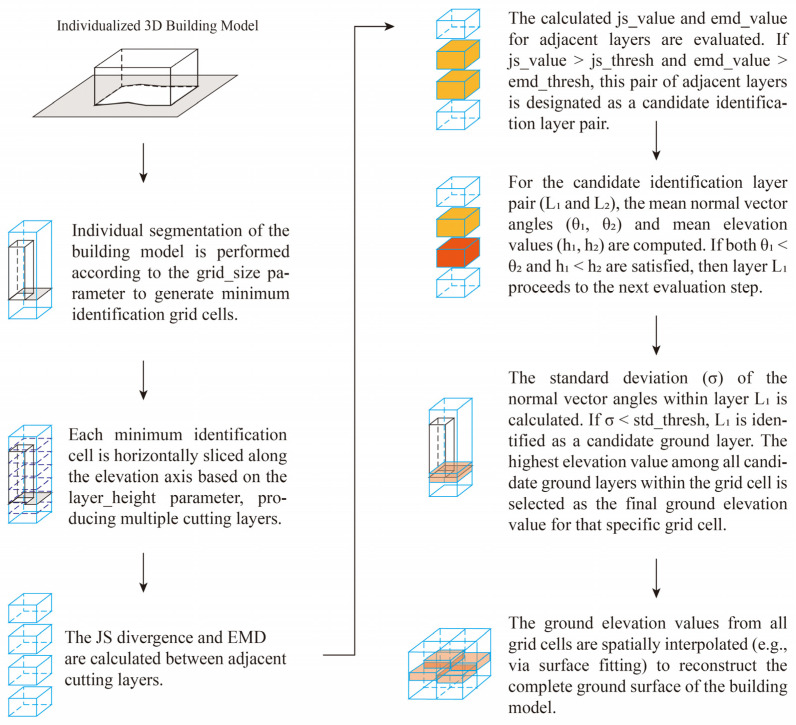
Workflow of the preliminary screening stage.

The coefficients are estimated using a least-squares approach, in which the design matrix $\left[x^{2},y^{2},xy,x,y,1\right]$ is constructed from the sample coordinates and solved to minimize the squared error between measured and predicted elevations. This process reconstructs a continuous and smooth ground-interface surface across the entire 3D scene, effectively interpolating elevation between grid locations. The reconstructed surface then serves as a reference baseline for ground determination: mesh faces whose elevations exceed the fitted surface by more than a specified tolerance are classified as non-ground elements, whereas those within the tolerance range are retained as ground. By integrating localized grid-based elevations through global polynomial surface fitting, the method enables identification and separation of ground and non-ground components, facilitating ground delineation in urban 3D models. The implementation of the method is shown in Figure 8 and Figure 9.

In real-world scenarios, building surfaces and their surrounding environments are often complex. The method described above may be insufficient for distinguishing between ground-like structures in close proximity to the ground, such as low vegetation or localized steep slopes. Therefore, building upon the aforementioned screening stage, we introduce a refinement stage based on geometric constraint consistency. This stage applies local geometric flatness constraints utilizing slope and roughness to eliminate irregular surface patches. Subsequently, global consistency constraints—including terrain fitting, connectivity, and elevation—are employed to remove regions that are fundamentally incompatible with the ground surface.

#### 2.2.2. Refinement Stage Based on Geometric Consistency

**Figure 10:**
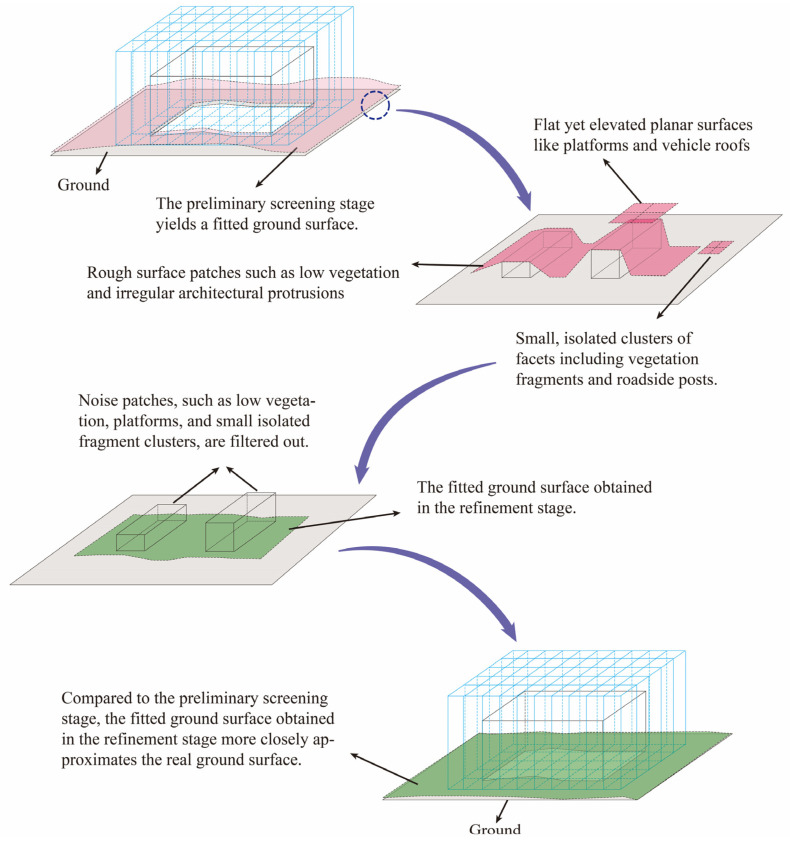
Schematic diagram of the refinement stage methodology.

During the preliminary screening stage, the algorithm derives elevation values proximate to the building-ground interface. However, segmentation accuracy is often compromised by interfering elements such as low vegetation and irregular architectural protrusions. To address this limitation, a refinement stage based on geometric consistency is introduced. The preliminary stage employs relaxed parameters to prioritize recall at a tolerable cost to precision. Subsequently, the refinement stage operates on this high-recall output, focusing on enhancing precision to achieve accurate segmentation. This stage improves precision through a dual approach: (1) enforcing local geometric flatness constraints via slope and roughness measures to eliminate noise patches (e.g., low vegetation); and (2) applying global consistency constraints—including terrain fitting, connectivity, and elevation coherence—to remove isolated patches that deviate from the inferred ground surface structure. The implementation of the method is shown in Figure 10.

##### Local Geometric Flatness Constraint

This constraint primarily targets local noise patches that persist after the preliminary screening stage, mainly comprising low vegetation and irregular protruding structures of buildings near the ground. These noise patches exhibit distinct spatial morphological characteristics compared to the flat state of the ground surface. Therefore, slope and roughness—effectively represented by the normal vector angle and its standard deviation per facet—serve as suitable filtering parameters.

Although these two morphological parameters are also utilized in the preliminary stage, their discriminatory objectives are fundamentally different. In the preliminary stage, the statistically derived mean normal vector angle and mean standard deviation are employed to rapidly identify the building-ground interface layer across large data volumes, achieving preliminary screening with high recall. However, as these statistics represent averages across entire candidate layers, they are insufficient for capturing localized noise within sub-regions of a layer.

In contrast, this refinement stage operates on individual facets. Following the preliminary screening, the computational load is significantly reduced, making facet-level analysis feasible. Consequently, in the refinement stage, the normal vector angle of each individual facet and its local neighborhood standard deviation are leveraged to perform precise filtering of the candidate data. The specific content is as shown in Table 3.

##### Global Structural Consistency Constraint

The local geometric flatness constraint primarily targets fine-scale noise patches, such as low vegetation and vehicles, by eliminating structures whose spatial morphology deviates from the ground. However, genuine ground data in most real-world scenarios typically forms several relatively large, spatially continuous surfaces. For planar structures near the ground—such as building base planes or vehicle roofs—there may exist facets that resemble the flat morphology of the ground but are not part of it. Consequently, further screening is required to eliminate such anomalous patches. Although these noise facets are morphologically similar to the ground, they exhibit significant discrepancies in global spatial and elevational consistency compared to the algorithm-fitted ground surface.

**Figure 11:**
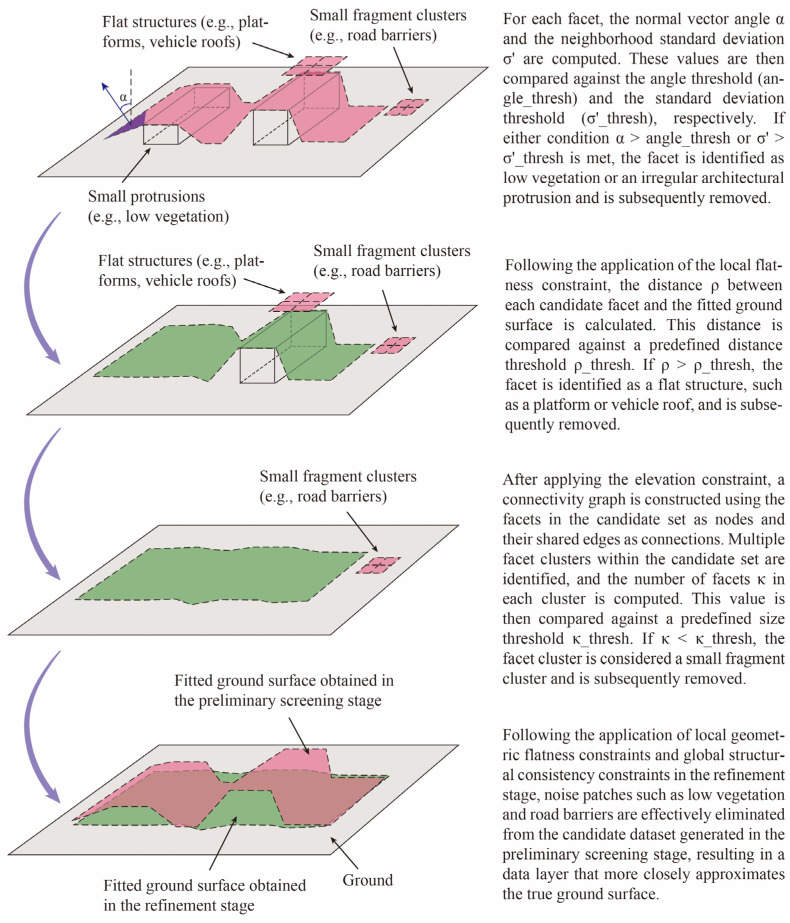
Workflow of the refinement stage.

Therefore, this stage introduces a global structural consistency constraint, implementing discrimination from three dimensions: terrain fitting, connectivity, and height. First, a surface model of the estimated ground is derived by fitting the candidate facets collectively, and the deviation of each candidate facet from this fitted ground is computed. Second, spatial connectivity analysis is performed to eliminate isolated clusters comprising only a small number of facets. Finally, a height constraint is applied to filter out elevated planar structures that cannot be distinguished by slope and roughness metrics alone. The implementation of the method is shown in Figure 11.

During the preliminary screening stage, parameters were appropriately relaxed to ensure high recall in identifying ground facets, albeit at the expense of reduced precision. In the subsequent refinement stage, while controlling the decline in recall through the application of the two aforementioned constraints, the precision of identification was significantly enhanced. Utilizing this two-stage ground recognition method based on spatial-geometric features, a candidate set of ground data was obtained. Finally, a fitting algorithm was applied to derive a fitted ground surface that more closely approximates real-world conditions. This surface was then employed to segment redundant ground components from the buildings, thereby achieving the objective of high-precision building individualization.

### 2.3. Experiment on Individual 3D Building Model Extraction

#### 2.3.1. Experimental Data and Environment

This study utilizes the SUM dataset to conduct the relevant experiments. The SUM dataset is a large-scale 3D mesh dataset developed by Delft University of Technology, The Netherlands. It covers an urban area of Helsinki, and the models were reconstructed using aerial oblique photography, offering high geometric accuracy and rich semantic annotation information [38]. In particular, the building and ground data account for a significant proportion of the entire dataset, exhibiting distinct spatial characteristics that provide well-suited data support for the proposed multiple spatial-geometric identification method. Therefore, the SUM dataset was selected as the experimental data. The experiments were carried out on a conventional PC environment (Intel Core i7 CPU @ 2.50 GHz, 32 GB RAM, Windows 11). The proposed methodology was implemented using the Python 3.10 programming language within the PyCharm platform 2024.3.1.1.

#### 2.3.2. Parameter Selection and Evaluation Metrics

The methodology proposed in this study is primarily divided into two stages. In the first stage, the SAMPolyBuild model is employed to perform semantic building recognition on the orthophotos derived from the oblique photogrammetric models, thereby achieving preliminary individualization of the models. This stage utilizes an existing image segmentation model, requiring no parameter configuration.

The second stage involves the identification and removal of ground noise data through the ground identification method integrating spatial-geometric features proposed in this paper. This stage mainly comprises two parts. The first part is the preliminary screening stage based on hierarchical features. Initially, the model data is spatially partitioned to obtain the minimum grid cells used as input for identification. This process primarily involves setting the grid segmentation size, specifically including the side length of segmentation units in the 2D plane and the interval height along the elevation direction. Concurrently, a lower threshold for the triangular facet count is set to ensure the algorithm operates with both efficiency and rationality. The JS divergence and EMD value are used to identify the interface structures between the ground and buildings. Subsequently, the mean normal vector angle and the standard deviation of normal vector angles are applied to recognize ground data. Due to variations in the characteristics of adjacent horizontal cutting layers across different grid cells, the JS divergence between layers is normalized prior to comparison to standardize the metric range during experiments. The EMD values are appropriately scaled to enhance the sensitivity of the discrimination threshold. Furthermore, a height tolerance is incorporated during discrimination to prevent misjudgments caused by elevation fluctuations in the model.

The second part is the refinement stage based on geometric consistency, which primarily refines the precision of the identification results from the preliminary screening stage. Redundant facets are filtered out through local geometric flatness constraints and global structural consistency constraints. The parameter settings used are summarized in Table 4 and Table 5.

In the methodological framework proposed in this study, achieving high-precision building individualization hinges on the accurate identification of ground data within the 3D model—a typical binary classification problem. Therefore, to evaluate the method’s performance, we adopt classic machine learning evaluation metrics based on the confusion matrix, which quantify classification accuracy across multiple dimensions to assess the method’s effectiveness and robustness. Accuracy represents the proportion of correctly classified triangular facets to the total number of facets, reflecting the overall correctness of the algorithm. Precision denotes the ratio of accurately identified ground facets to all facets predicted as ground, measuring the exactness of the ground identification. Recall refers to the proportion of actual ground facets that are correctly identified, indicating the algorithm’s ability to cover all relevant instances. The F1-score, as the harmonic mean of Precision and Recall, provides a comprehensive measure that balances both metrics.

## 3. Results

### 3.1. Experiments and Results

**Figure 12:**
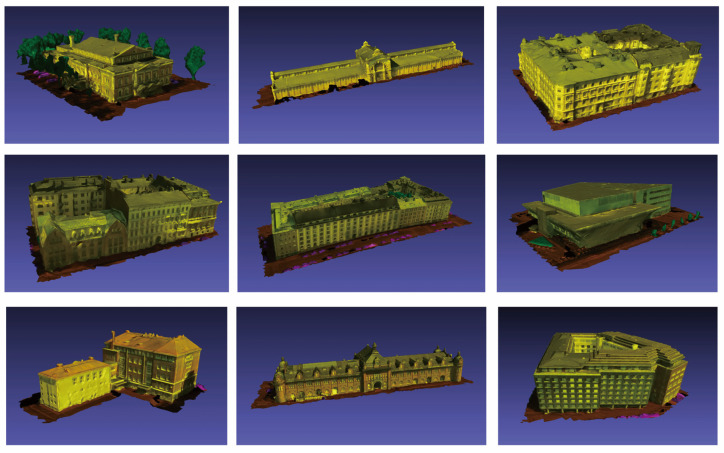
Partial dataset of selected building models for the experiment.

To validate the effectiveness of the building individualization method proposed in Section 2, we selected 20 models from the SUM dataset that are representative in terms of spatial structure to conduct the experimental validation. A subset of the experimental data is illustrated in Figure 12. The selected models encompass the main structural characteristics of the buildings within the dataset, primarily featuring Neoclassical architectural styles, thereby providing a comprehensive evaluation of the method’s capability in extracting ground data.

The proposed method was applied to perform ground identification on the selected building models, with the results summarized in Table 6.

Based on the result data, the method proposed in this paper demonstrates high accuracy for ground identification across all tested models, with values exceeding 90%—reaching a maximum of 98.37% and an average of 95.83%. This indicates that the method performs accurately in the global judgment of the majority of facets. The precision metric is largely above 85%, peaking at 98.50% with an average of 87.82%, suggesting that approximately 88% of the facets identified as ground are true ground facets, reflecting a low false positive rate and effective removal of pseudo-ground surfaces such as platforms and vehicle roofs. The recall rate mostly exceeds 80%, with a maximum of 88.05% and an average of 80.06%, implying that around 80% of the actual ground data can be correctly identified, while about 20% remains undetected. The F1-score nearly consistently surpasses 0.8, reaching a maximum of 0.88 and averaging 0.83, indicating that the method maintains a high coverage of ground facets while preserving accurate discrimination, resulting in a relatively balanced overall performance.

**Figure 13:**
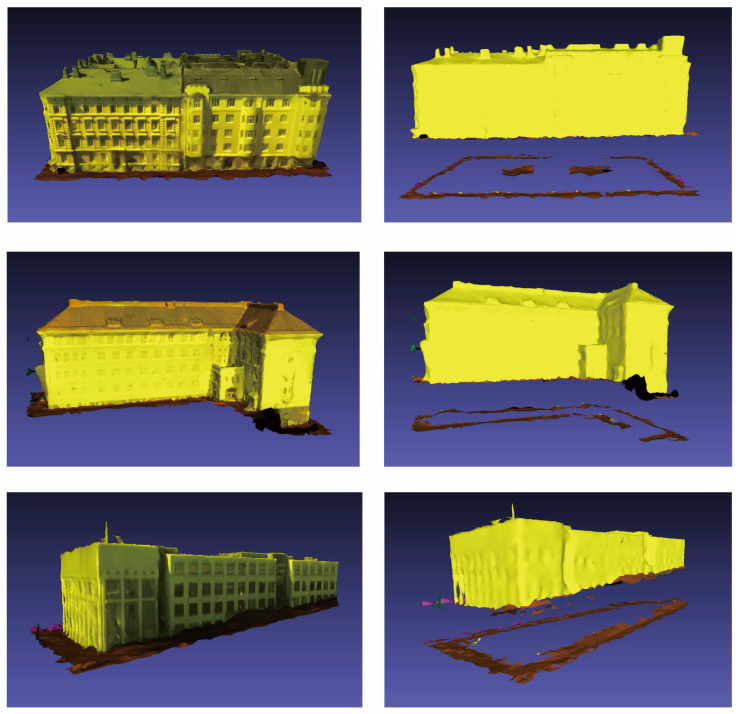
Partial experimental segmentation results.

To demonstrate the segmentation effectiveness, three typical cases from the experiments were selected for presentation, as shown in Figure 13. The left side of each subfigure displays the original morphology of the model, while the right side shows the results of ground identification and segmentation achieved by applying the proposed method. In the visualization, yellow indicates building data, brown represents ground data, and pink denotes vehicles. The first model features a regular architectural structure, whereas the second and third models possess inclined curved surfaces at their base, with complex structural details near the ground. The segmentation results demonstrate that the proposed method effectively identifies and separates the ground data.

### 3.2. Comparative Experiments

**Figure 14:**
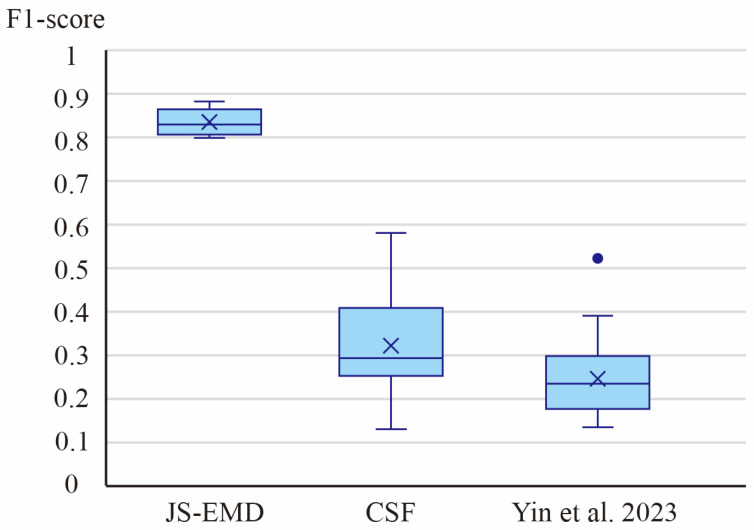
Box plots of the comparative experiments [40].

To validate the effectiveness of the proposed method, comparative experiments were conducted on the same dataset. Since the proposed method primarily combines an unsupervised approach with spatial-geometric features, representative methods based on traditional spatial-geometric features were selected to ensure comparability. The Cloth Simulation Filter (CSF) is a classic algorithm for ground data extraction [39], initially proposed by Zhang et al. Its fundamental concept involves inverting the model, placing an initially flat virtual cloth above it, and allowing the cloth to descend and deform under gravity until it makes contact with the model—the contacted data is then classified as ground. Similarly, Yin et al. [40] also achieves 3D model individualization through hierarchical segmentation and spatial-geometric features, sharing conceptual similarities with the method proposed herein. Therefore, employing these two methods as baselines is meaningful for comparison. The experimental results of the three methods are presented using box plots, as illustrated in Figure 14.

Analysis of the box plots reveals that the proposed method outperforms both the traditional geometric feature-based algorithm and the cloth simulation filter across all 20 experimental datasets. The proposed method achieves a median F1-score of approximately 0.84, exhibits the smallest interquartile range (IQR), and displays the shortest whiskers. This indicates that the proposed method attains the highest recognition accuracy, the least variation, and the strongest robustness across models with different structural characteristics. In contrast, the traditional geometric feature-based algorithm and the cloth simulation filter yield median F1-scores of about 0.24 and 0.30, respectively. Their distributions are widely dispersed, with relatively longer whiskers, suggesting that these methods are more sensitive to the diverse structural types within the experimental data and demonstrate significantly lower recognition accuracy.

### 3.3. Ablation Study

**Figure 15:**
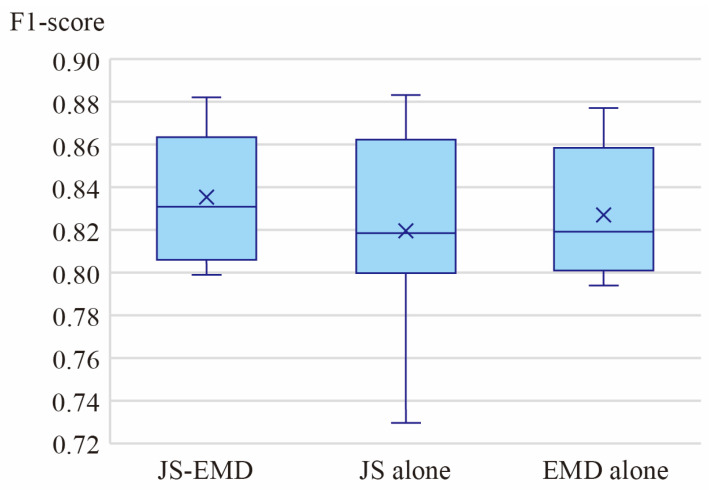
Box plots of the ablation study.

In the proposed method, the JS-EMD metric represents statistically significant parameters. Compared to other traditional geometric parameters, the combined application of these two indices in hierarchical statistical discrimination necessitates validation of their necessity. Therefore, we designed an ablation study to compare the experimental results under three conditions: using only the JS divergence, using only the EMD, and using the combined JS-EMD metric. This aims to verify the complementary hypothesis that “JS measures shape difference while EMD measures overall displacement.” The evaluation was conducted on the same dataset with fixed hyperparameters, and the resulting F1-scores are presented in Figure 15.

Analysis of the box plots indicates that the joint JS-EMD metric outperforms the methods using either JS divergence or EMD alone, in terms of both mean and median values. Furthermore, it exhibits a smaller interquartile range (IQR) and shorter whiskers, demonstrating higher stability. The method employing only JS divergence shows prominent lower outliers (approximately 0.73), indicating greater result variability. Although the method using only EMD does not exhibit significant lower outliers, it is inferior to the joint JS-EMD metric in both box width and median value.

## 4. Discussion

In Section 3, we conducted experiments using the proposed method, alongside comparative and ablation studies to validate its effectiveness and stability. Following the experiments on the 20 representative individual building models selected from the SUM dataset, the average results obtained are as follows: accuracy of 95.83%, precision of 87.82%, recall of 80.06%, and an F1-score of 0.8349. The experimental results indicate that the proposed method achieves favorable identification performance in the process of ground extraction from 3D models, with high overall correctness and identification precision.

This performance stems from the proposed two-stage identification method based on spatial-geometric features. In the preliminary screening stage, parameters are appropriately relaxed to ensure high recall. In the subsequent refinement stage, the candidate set identified in the previous stage is corrected, thereby enhancing precision with minimal reduction in recall.

Compared to accuracy and precision, the recall rate is somewhat lower, indicating a relatively conservative nature of the method. This aligns with its core principle: leveraging facet geometric morphology and topological constraints effectively suppresses false ground data, thereby improving precision. However, this approach may lead to missed detection of true ground data in scenarios involving sloped buildings or complex near-ground structures, resulting in a lower recall rate. This trade-off effectively mitigates Type I errors, ensuring the integrity of the building data after individualization.

To ensure an objective evaluation of the proposed method, we analyzed the two models with the lowest F1-scores in the experiments, namely Building No. 7 and Building No. 20. Their segmentation accuracy is detailed in Table 7.

**Figure 16:**
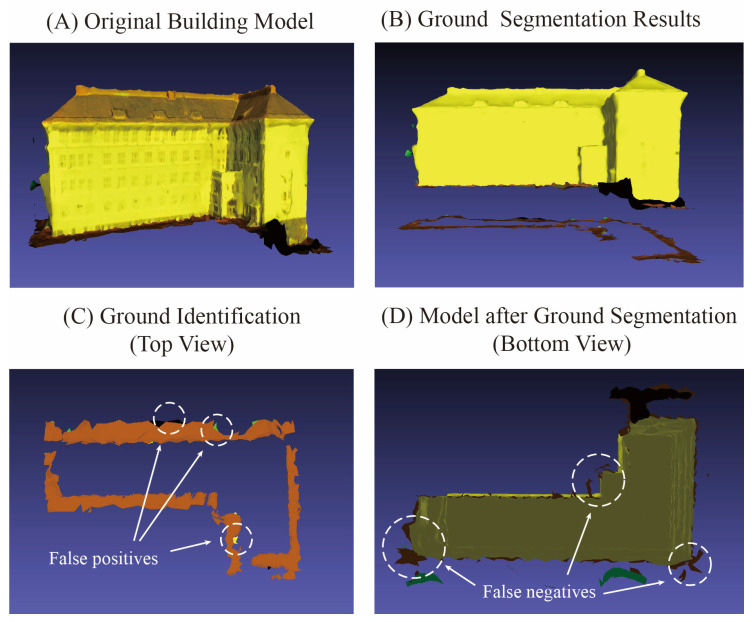
Segmentation results of building No. 7.

Analysis of the results indicates that the lower F1-scores for both models are primarily attributable to their relatively low recall rates. In particular, the recall for Building No. 7 falls below 70%, while its precision remains high. This suggests that although the ground data identified by the method is almost entirely correct, a significant number of true ground instances are missed. For Building No. 20, both precision and recall are approximately 80%, indicating a more balanced occurrence of false positives and false negatives. The segmentation results for both buildings are visualized in Figure 16.

As observed from the visualization results, the method demonstrates high precision in identifying ground data. In Figure 16C, only a small number of individual facets are misidentified as ground. In Figure 16D, however, there is a portion of missed ground data, particularly noticeable in the lower-left region where the ground data exhibits a stacked configuration in three-dimensional space. Due to the irregular folded morphology of the building model in this area, the stacked ground data is prone to being misclassified as part of the surrounding building structure during the ground identification process, resulting in a higher rate of missed detections. Additionally, the overall quantity of ground data in this model is limited, meaning that even a small number of missed instances can lead to a significant reduction in the recall rate.

**Figure 17:**
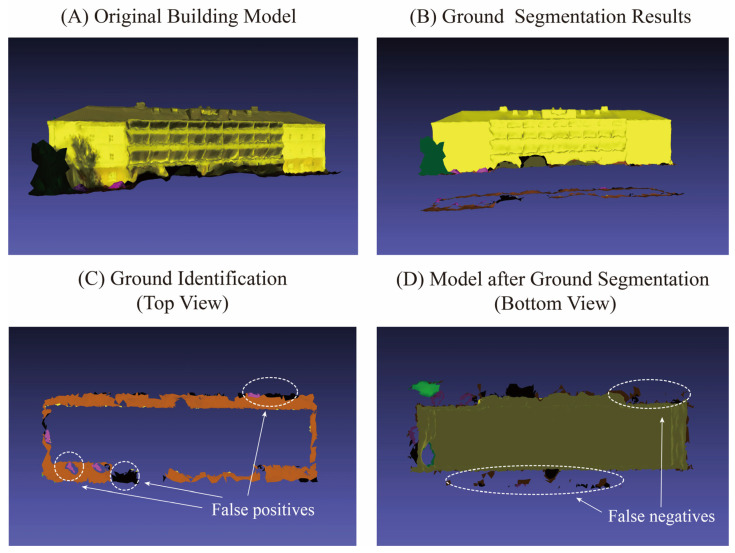
Segmentation results of building No. 20.

For Model No. 20 in Figure 17, the occurrences of false positives and false negatives in ground identification are relatively balanced. In Figure 17C, the false positives primarily consist of pink vehicle roof data and black unclassified data. The vehicle roofs exhibit a relatively flat morphology, and the base of this building is inclined, causing the elevation of the vehicle roof data to approximate that of the adjacent ground surface on one side, leading to misidentification. The black unclassified data, overall, resembles the ground in shape, resulting in false detection. In Figure 17D, the missed detections are predominantly concentrated on the frontal ground data that is distant from the building façade. Due to the complex structural morphology in this region, the surface patches are irregular. Distant ground data cannot be effectively distinguished based on geometric features alone, making it prone to being missed.

In the comparative experiments, the method proposed in this paper was compared with traditional geometric feature-based algorithms and the cloth simulation filter. The F1-score box plots of the experimental results demonstrate that the proposed method significantly outperforms these two baseline algorithms. This superiority primarily stems from the fact that, after the preliminary individualization of the model, the proposed method further performs a two-stage structural identification through spatial partitioning of the model. This decomposes the complex and variable model structures into a collection of single, simple spatial structures, allowing the discrimination to focus more on the specific characteristics of each sub-model. In contrast, both the cloth simulation filter and traditional spatial-geometric algorithms perform calculations based on the entire model. Parameter adjustments from a global perspective are relatively insufficient for capturing local detailed structures, which explains the superior performance of the proposed method in ground data identification.

Within the proposed method, the innovative introduction of two statistical parameters—The JS divergence and Earth Mover’s Distance—for identifying architectural spatial-geometric morphology is noteworthy. Other parameters involved have been referenced in previous methods. Therefore, in the ablation study, we primarily investigated the ground identification performance under three modes: using JS divergence alone, using EMD alone, and using the combined JS-EMD metric, to validate the necessity of their joint application. Analysis of the F1-score box plots from the experimental results indicates that the combined JS-EMD metric surpasses the use of either parameter alone in terms of both mean and median values, while also exhibiting a smaller box size and shorter tails, suggesting greater stability.

This improvement is attributed to the complementary nature of the two metrics. JS divergence is sensitive to local abrupt changes, whereas EMD more effectively captures shifts in the overall angular distribution. Consequently, when the primary difference between two segmentation layers is an overall angular translation—manifested in the histograms as similar peak shapes with only a positional shift—the increase in JS divergence tends to be small and may fall below the threshold, leading to missed detection. Conversely, when localized sharp peaks appear in the histogram—occupying a small area proportion but spanning a wide angular range—the EMD parameter accumulates linearly with distance and can be easily amplified, resulting in false positives. Leveraging their complementary roles, the joint criterion is adopted: only when both parameters simultaneously satisfy their respective thresholds do we conclude that a significant change in the distribution of facet normal vector angles has occurred between adjacent segmentation layers, thereby successfully capturing the abrupt transition layer from ground to building façade.

The experimental results and analysis presented above demonstrate that the proposed method can effectively individualize oblique photogrammetric 3D models with high recognition accuracy. The main contributions of this work are threefold:

1. To address the inefficiency of manual building individualization in current 3D modeling workflows, this paper proposes a novel building individualization method that integrates the SAMPolyBuild model with spatial-geometric features. By interpreting building orthoimagery, the method extracts the horizontal projection boundaries of buildings. Compared to algorithms that operate directly on 3D models, the proposed approach is more lightweight and straightforward to implement.
2. To tackle the problem of redundant ground data in building models, we developed a two-stage ground identification method that integrates multiple spatial-geometric features. This approach utilizes characteristics of triangular facet normal vector angles across both local and global scales to identify ground data within building models, effectively capturing structural details and achieving high recognition accuracy.
3. Compared to current mainstream deep learning models, the proposed method fully capitalizes on the zero-shot or few-shot learning capabilities of the Segment Anything Model (SAM), resulting in stronger generalization across various building types. This significantly reduces the heavy reliance on extensive pre-training of deep learning models and substantially improves the efficiency of building individualization in practical applications.

Despite its promising performance, the proposed method shows limitations when dealing with highly complex building structures. The main shortcomings are as follows:

1. Parameter selection—such as grid size and layer height—is highly dependent on the spatial configuration of the building model. Optimal parameters vary across different structures, indicating a need for an adaptive parameter-setting mechanism to improve recognition efficiency;
2. The method exhibits sensitivity to noise, such as complex architectural detail structures, particularly when these elements are located near the ground. Future work will incorporate additional features (e.g., texture information) to enhance robustness and accuracy.

## 5. Conclusions

In the construction of urban 3D models, structural individualization is essential for enabling efficient data application and management. Addressing the practical need for individualizing oblique photogrammetric 3D models, this paper proposes a novel building individualization method integrating the SAMPolyBuild model with multiple spatial-geometric features. The procedure consists of three main steps: first, buildings are identified and extracted from orthophotos of the model area using SAMPolyBuild; then, the extracted 2D boundaries are used to guide the cutting of the 3D model; and finally, a dual-stage, multiple spatial-geometric feature-based identification method is employed to remove residual ground elements, thereby achieving precise individualization of the 3D models.

Experiments and comprehensive analysis conducted on the SUM dataset demonstrate that the proposed method achieves high-precision building extraction, offering a viable and effective solution for the individualization of oblique photogrammetric 3D models. By leveraging the strong segmentation capabilities of the Segment Anything Model, the method successfully extends image-based segmentation concepts to 3D spatial modeling. Although the current approach relies solely on orthoimagery for building boundary extraction—which may affect accuracy in complex scenarios—it establishes a meaningful direction toward large-scale 3D segmentation with minimal training overhead. Future work will explore integration of multi-angle imagery-based segmentation and fusion with additional features to further improve segmentation accuracy, paving the way toward fully automated individualization of oblique photogrammetric models.

## Figures and Tables

**Table 1:** Values of JS divergence for normal vector angle distributions between adjacent cutting layers in the model.

Cutting Layers	JS Divergence
Layers 1–2	0.179274
Layers 2–3	0.059801
Layers 3–4	0.172501
Layers 4–5	0.017629
Layers 5–6	0.226602
Layers 6–7	0.105232
Layers 7–8	0.230292

**Table 2:** Earth Mover’s Distance (EMD) values for normal vector angle distributions between adjacent cutting layers in the model.

Cutting Layers	JS Divergence
Layers 1–2	34.669285
Layers 2–3	7.390554
Layers 3–4	36.918021
Layers 4–5	2.537385
Layers 5–6	41.590462
Layers 6–7	24.637143
Layers 7–8	15.615

**Table 3:** Functional comparison of normal vector parameters in the two-stage method.

	Preliminary Screening Stage (Hierarchical Statistics)	Refinement Stage (Local Region)
Parameters	Mean and standard deviation of facet normal vectors per layer	Normal vector of individual facet and standard deviation within its neighborhood
Objective	High recall: rapidly identifies near-ground layers	High precision: eliminates false positives
Statistical Granularity	Grid elevation layer	Individual facet and its neighborhood
Outcome	Reduces computational space and global fitting cost	Corrects fitting deviation and improves segmentation accuracy

**Table 4:** Parameter settings for the preliminary screening stage.

Parameter	Name	Value	Description
Cell_size	Cell Size	30 m	Cell dimension used for algorithmic identification
layer_height	Layer Height	2 m	Height interval for horizontal slicing
height_ratio	Identification Height Ratio	0.1	Ratio to limit the vertical recognition range for improved computational efficiency
min_faces_per_grid_cell	Minimum Facet Count per Grid Cell	10	Minimum number of facets required within a grid cell to execute the algorithm
js_thresh	JS Divergence Threshold	0.4	Threshold for Jensen-Shannon divergence, used jointly with EMD to detect structural changes
emd_thresh	EMD Threshold	10	Threshold for Earth Mover’s Distance, used jointly with JS divergence to detect structural changes
std_thresh	Normal Vector Angle STD Threshold	30°	Threshold constraining the spatial smoothness (standard deviation) of normal vector angles
height_offset	Height Tolerance	1 m	Allowable tolerance in elevation judgment within a layer

**Table 5:** Parameter settings for the refinement stage.

Parameter	Name	Value	Description
angle_max_deg	Slope Angle Threshold	35°	Constraint value for the facet slope angle
rough_std_max_deg	Normal Vector Angle Standard Deviation Threshold	25°	Constraint value for the standard deviation of normal vector angles within the neighborhood (indicating roughness)
plane_dist_max	Relative Height Threshold to the Ground	0.6	Unilateral decision threshold for the distance between a facet and the fitted ground surface
min_comp_faces	Minimum Face Count per Connected Cluster	30	Filtering constraint based on connected component size

**Table 6:** Experimental results.

Building No.	Accuracy	Precision	Recall	F1
1	0.9550	0.8652	0.7581	0.8081
2	0.9370	0.7372	0.8805	0.8025
3	0.9673	0.8830	0.8757	0.8793
4	0.9811	0.8844	0.8433	0.8634
5	0.9043	0.9437	0.7755	0.8514
6	0.9700	0.9041	0.7247	0.8046
7	0.9552	0.9850	0.6717	0.7987
8	0.9556	0.9378	0.8260	0.8784
9	0.9676	0.8864	0.8031	0.8427
10	0.9577	0.8133	0.8002	0.8067
11	0.9523	0.9239	0.8092	0.8627
12	0.9837	0.9220	0.8452	0.8820
13	0.9722	0.8991	0.7581	0.8226
14	0.9604	0.9075	0.8312	0.8677
15	0.9492	0.9216	0.7153	0.8054
16	0.9795	0.8907	0.8349	0.8619
17	0.9560	0.8311	0.8473	0.8391
18	0.9449	0.8117	0.8093	0.8105
19	0.9590	0.8123	0.8091	0.8107
20	0.9588	0.8048	0.7955	0.8001
Mean	0.9583	0.8782	0.8007	0.8349

**Table 7:** Results of low-performance experiments.

Building No.	Accuracy	Precision	Recall	F1
7	0.9552	0.9850	0.6717	0.7987
20	0.9588	0.8048	0.7955	0.8001

## Data Availability

The Semantic Urban Meshes (SUM) dataset employed in this study is publicly accessible and was utilized to evaluate the reliability of the proposed algorithm. This large-scale annotated 3D mesh dataset can be freely downloaded from its official project page hosted by Delft University of Technology: https://3d.bk.tudelft.nl/projects/meshannotation/, accessed on 27 April 2025.
